# Changes in retinal venous diameter in proliferative diabetic retinopathy patients with diabetic macular edema following faricimab treatment: an observational study

**DOI:** 10.3389/fendo.2026.1837934

**Published:** 2026-07-09

**Authors:** Hui Zhao, Yuanqing Liu, Lingchao Zhang, Xiaonan Zhao, Nan Wang, Yuan Tao, Hong Wang

**Affiliations:** 1 Department of Ophthalmology, Jinan Second People’s Hospital, Jinan, China; 2 Department of Ophthalmology, Jinan Huashi Ophthalmology Hospital, Jinan, China; 3 Department of Ophthalmology, Qilu Hospital of Shandong University, Jinan, China; 4 Department of Ophthalmology, Jinan Aier Eye Hospital, Jinan, China

**Keywords:** diabetic macular edema, faricimab, hard exudates, microaneurysm, neovascularization area, proliferative diabetic retinopathy, retinal venous diameter

## Abstract

**Introduction:**

To evaluate the effect of intravitreal faricimab injections on retinal venous diameter in proliferative diabetic retinopathy (PDR) patients with diabetic macular edema (DME).

**Methods:**

This single-center, retrospective cohort study included 46 PDR patients (46 eyes) with DME, treated between August 2024 and January 2025. All participants received intravitreal faricimab combined with panretinal photocoagulation (PRP). Best-corrected visual acuity (BCVA, logMAR), central foveal thickness (CFT, μm), microaneurysm (MA) count, hard exudates (HE), neovascularization (NV) area, and retinal venous diameter were assessed at baseline and at 1, 3, and 6 months post-treatment.

**Results:**

Significant improvements in BCVA were observed at 1, 3, and 6 months (0.52 ± 0.16, 0.48 ± 0.18, 0.40 ± 0.15 vs. baseline 0.66 ± 0.18; all P < 0.05). CFT showed a significant reduction at all time points (381.35 ± 40.75, 327.30 ± 43.40, 297.56 ± 35.81 μm vs. baseline 472.34 ± 47.23 μm; all P < 0.05). The MA count showed no significant reduction at 1 month (P > 0.05) but decreased significantly thereafter (72.71 ± 20.53 and 63.06 ± 17.08 at 3 and 6 months, respectively; both P < 0.05). Reductions in HE area were not significant at 1 or 3 months (in 2 and 6 patients, respectively; both P > 0.05). At 6 months, a significant reduction in HE area was observed in 14 patients (P < 0.05). The NV area was significantly reduced at 1, 3, and 6 months compared to baseline (0.17 ± 0.10 mm², 0.17 ± 0.09 mm², and 0.17 ± 0.08 mm² vs. 0.98 ± 0.17 mm²; all P<0.05). Retinal venous diameter showed no significant change at 1 and 3 months compared with baseline (both P > 0.05) but was significantly reduced at 6 months (299.40 ± 19.56 μm; P < 0.05).

**Conclusion:**

Intravitreal faricimab was safe and effective for PDR patients with DME. A trend toward morphological reversal of retinal venous diameter was demonstrated at 6 months, suggesting that faricimab may influence retinal microvascular remodeling through dual inhibition of the VEGF-A and Ang-2 pathways.

## 1 Introduction

Diabetic retinopathy (DR) is one of the most common and vision-threatening microvascular complications of diabetes mellitus (1). It represents the leading cause of blindness among the global working-age population (2, 3). Diabetic macular edema (DME) is a critical pathological manifestation during DR progression, characterized by fluid accumulation within the macular and thickening of the neurosensory retina due to abnormally increased retinal vascular permeability (4, 5). If left untreated, DME can result in irreversible central vision loss (4, 5). Intravitreal anti–vascular endothelial growth factor (anti-VEGF) therapy has been established as the first-line treatment for DME (6). Faricimab is a novel bispecific antibody that simultaneously inhibits both VEGF-A and angiopoietin-2 (Ang-2) (7). It is the first dual-target inhibitor specifically designed for intravitreal administration and approval for clinical application. Two-year follow-up data from the phase III YOSEMITE and RHINE randomized controlled trials demonstrated that faricimab was non-inferior to aflibercept in improving best-corrected visual acuity (BCVA), reducing central foveal thickness (CFT), and sustaining long-term efficacy, with comparable safety profiles (8).

This study aimed to systematically evaluate the multidimensional efficacy of intravitreal faricimab combined with panretinal photocoagulation (PRP) in proliferative diabetic retinopathy (PDR) patients with DME. A primary focus was the quantitative assessment of alterations in retinal venous diameter, a key morphological parameter reflecting retinal hemodynamic status and vascular structural stability. Concurrently, we assessed the dynamic evolution of classical functional and structural biomarkers, including BCVA, CFT, microaneurysm (MA) count, hard exudate (HE) area, and neovascularization (NV) area.

## 2 Materials and methods

### 2.1 Study population

This study consecutively enrolled patients diagnosed with PDR at the Department of Ophthalmology, Qilu Hospital of Shandong University, between August 2024 and January 2025. The diagnosis of PDR was confirmed based on fundus examination, fundus fluorescein angiography (FFA), and optical coherence tomography (OCT) (**Table 1**). Inclusion criteria were as follows: (1) diagnosis of PDR according to established International Clinical classification criteria; (2) a history of type 2 diabetes mellitus with glycated hemoglobin (HbA1c) <10% and blood pressure <160/90 mmHg under standardized systemic management; (3) no history of any prior retinal laser photocoagulation, intravitreal injections of anti-VEGF, or intravitreal corticosteroid injections. The exclusion criteria were: (1) type 1 diabetes mellitus; (2) Presence of significant media opacities (e.g., dense vitreous hemorrhage) that precluded acquisition of high-quality, gradable FFA or OCT images essential for accurate assessment; (3) concomitant non-diabetic retinal vascular diseases. This study was conducted in accordance with the tenets of the Declaration of Helsinki and received approval from the Medical Ethics Committee of Jinan Second People’s Hospital (Approval No.: JNEYE20260603).

**Table 1:** Baseline clinical characteristics of patients.

Characteristic	Value
Gender, n (%)	
Male	24(52.2%)
Female	22(47.8%)
Mean age ± SD(years)	60.73 ± 6.56
Study Eye, n (%)	
Right eye	23(50.0%)
Left eye	23(50.0%)
Mean BCVA ± SD (Log MAR)	0.66 ± 0.18
Mean CFT ± SD (μm)	472.34 ± 47.23
Mean Duration of Diabetes ± SD (years)	5.36 ± 1.95

SD, standard deviation; BCVA, best-corrected visual acuity; CFT, central foveal thickness; log MAR, logarithm of the minimum angle of resolution.

### 2.2 Methods

We retrospectively collected baseline and follow-up data from the electronic medical record (EMR) system including age, sex, laterality of the involved eye, BCVA, CFT(μm), MA count, HE area (mm²), NV area (mm²), and retinal venous diameter (μm). All treatment-related adverse events were systematically documented. Changes in these parameters were compared between baseline and at 1, 3, and 6 months post-treatment. All image assessments were performed independently by two experienced retinal specialists, and discrepancies were resolved by consensus.

The treatment regimen initiated with a loading phase consisting of four monthly injections (the “4 + PRN” protocol). All procedures were administered by the same experienced ophthalmologist. Prophylactic topical levofloxacin was administered four times daily for 3 days prior to each injection. PRP was performed within one week following the first faricimab injection. For recurrent DME during follow-up, standardized retreatment was initiated according to the Early Treatment Diabetic Retinopathy Study (ETDRS) guidelines.

BCVA was assessed using the Early Treatment Diabetic Retinopathy Study (ETDRS) chart and all values were converted to logarithm of the minimum angle of resolution (logMAR) for statistical analysis. FFA was performed using a Heidelberg SPECTRALIS HRA fundus camera and video angiography system. OCT imaging was conducted with the Cirrus HD-OCT device (Carl Zeiss Meditec), and all subsequent quantitative analyses utilized the manufacturer’s proprietary software. CFT was defined as the vertical distance from the internal limiting membrane (ILM) to the retinal pigment epithelium (RPE) at the foveal center. Three measurements were obtained for the same eye at each follow-up time point, and the mean value was recorded as the CFT. All measurements were performed by two experienced retinal specialists. Pupils were dilated with 1% tropicamide eye drops administered 15 minutes before examination. Standard FFA was performed in all patients, and images were archived for subsequent analysis.

The central retinal vein equivalent (CRVE) was derived from FFA images. Images centered on the macula and optic disc were selected, and all venules coursing through an annular zone located 1.0 to 1.5 disc diameters from the optic disc margin were measured. The diameters of the six largest venules were measured and integrated using the Parr–Hubbard formula and modified by Knudtson. CRVE represents the equivalent diameter of the central retinal vein in a two-dimensional projection (9, 10). MA counting and NV area were assessed on 55°field of view FFA images centered on the fovea. HE area was identified and quantified using color fundus photographs acquired with a VISUCAM 224 fundus camera and were assisted by professional image processing software.

### 2.3 Statistical analysis

All statistical analyses were conducted using IBM SPSS Statistics software (version 25.0). Continuous variables are presented as mean ± standard deviation (SD), whereas categorical variables are expressed as percentages (%). Paired t-tests were used to compare normally distributed continuous data, while non-parametric tests were applied for continuous data that violated the normality assumption. Categorical data were compared using the chi-square test. A two-sided P-value of less than 0.05 was considered indicative of statistical significance.

## 3 Results

### 3.1 Baseline patient characteristics

A total of 46 PDR patients (46 eyes) with DME were included, comprising 24 males (24 eyes) and 22 females (22 eyes). The mean age was 60.73 ± 6.56 years, and the mean duration of diabetes was 5.36 ± 1.95 years (**Table 1**).

### 3.2 Changes in BCVA (logMAR) and CFT

**Figure 1:**
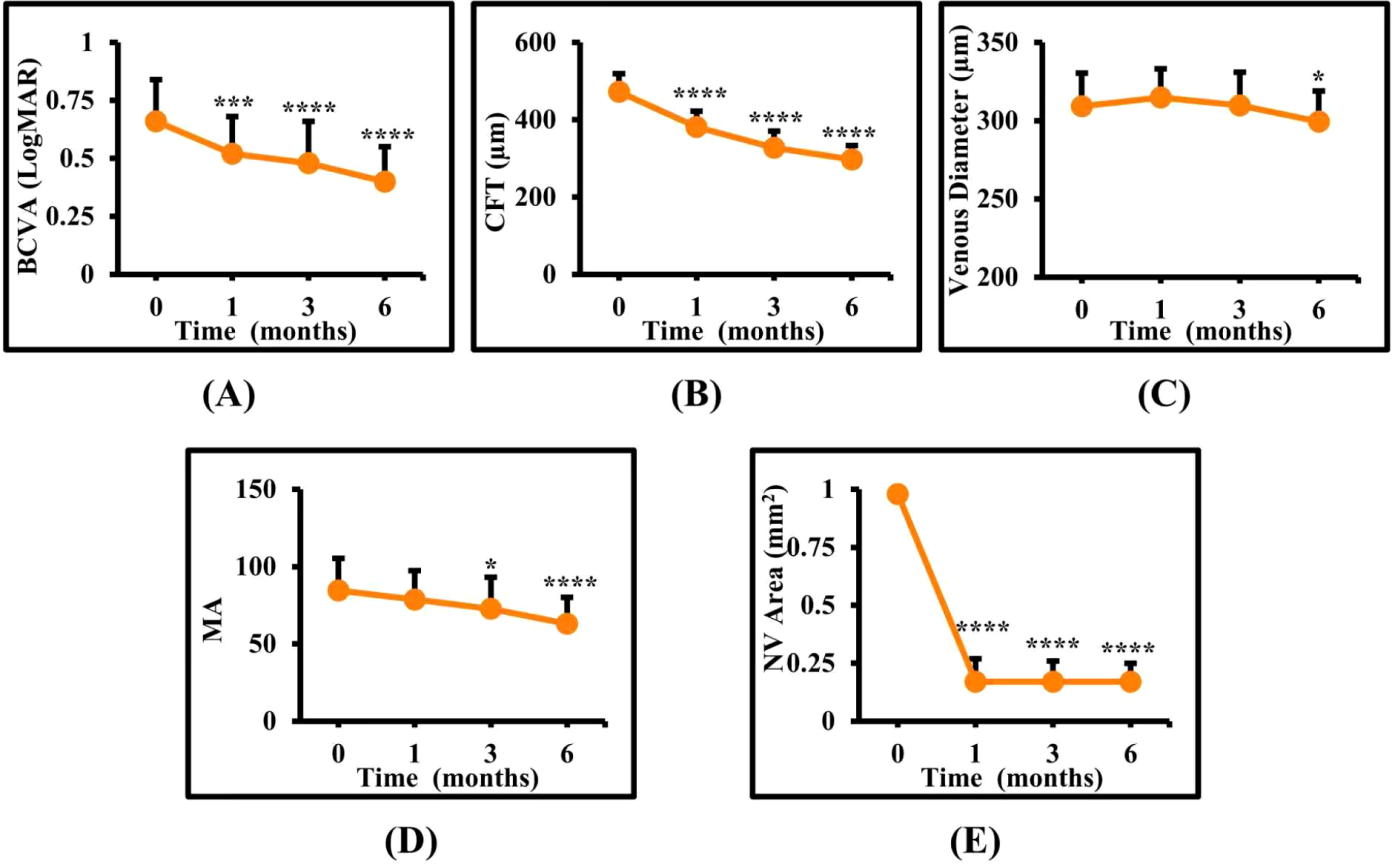
Changes in the BCVA (logMAR) **(A)**, CFT (μm) **(B)**, retinal vein diameter (μm) **(C)**, number of MA **(D)**, NV (mm^2^) **(E)**, at different time points before and after treatment. BCVA, best-corrected visual acuity. CFT, central foveal thickness. MA, microaneurysm. NV, neovascularization. *P < 0.05 vs.pretreatment, ***P < 0.001 vs. pretreatment, ****P < 0.0001 vs. pretreatment.

BCVA (logMAR) demonstrated significant improvement from baseline (0.66 ± 0.18) at all follow-up visits (0.52 ± 0.16 at 1 month, 0.48 ± 0.18 at 3 months, and 0.40 ± 0.15 at 6 months; all P < 0.05). CFT decreased significantly from baseline (472.34 ± 47.23 μm) to 381.35 ± 40.75 μm at 1 month, 327.30 ± 43.40 μm at 3 months, and 297.56 ± 35.81 μm at 6 months (all P < 0.05) (**Tables 2**, **3**; **Figures 1A, B**).

**Table 2:** Comparison of BCVA (LogMAR) before and after treatment.

Parameter	Before treatment	1 Month post-treatment	3 Months post-treatment	6 Months post-treatment
n=46	0.66 ± 0.18	0.52 ± 0.16	0.48 ± 0.18	0.40 ± 0.15
t-value		3.8352	4.6161	7.6617
P-value		<0.001	<0.001	<0.001

**Table 3:** Comparison of CFT (μm) before and after treatment.

Parameter	Before treatment	1 Month post-treatment	3 Months post-treatment	6 Months post-treatment
n=46	472.34 ± 47.23	381.35 ± 40.75	327.30 ± 43.40	297.56 ± 35.81
t-value		9.8935	15.3361	19.9989
P-value		<0.001	<0.001	<0.001

### 3.3 Changes in retinal venous diameter

Retinal venous diameter showed no significant change at 1 month (314.92 ± 18.31 μm) or 3 months (309.81 ± 21.19 μm) compared with baseline (309.26 ± 21.11 μm; both P > 0.05), but a significant reduction was observed at 6 months post-treatment (299.40 ± 19.56 μm; P < 0.05) (**Table 4**; **Figure 1C**).

**Table 4:** Comparison of retinal venous diameter (μm) before and after treatment.

Parameter	Before treatment	1 Month post-treatment	3 Months post-treatment	6 Months post-treatment
n=46	309.26 ± 21.11	314.92 ± 18.31	309.81 ± 21.19	299.40 ± 19.56
t-value		1.3750	0.1277	2.3221
P-value		0.1726	0.8987	0.0225

### 3.4 Changes in MA, HE and NV area

The MA count showed no significant change at 1 month (78.70 ± 18.66 vs 84.60 ± 20.74 at baseline; P > 0.05), but decreased significantly at 3 months (72.71 ± 20.53) and 6 months (63.06 ± 17.08) (both P < 0.05). A reduction in HE area was observed in 2 patients at 1 month and 6 patients at 3 months, with no statistically significant difference observed (P > 0.05). By 6 months, the number of patients showing HE reduction increased to 14, achieving statistical significance (P < 0.05). NV area decreased significantly from baseline (0.98 ± 0.17 mm²) to 0.17 ± 0.10 mm², with 0.17 ± 0.09 mm², and 0.17 ± 0.08 mm² at 1, 3, and 6 months, respectively, and the comparisons with baseline at each time point were statistically significant (all P < 0.05) (**Tables 5**–**7**; **Figures 1D, E**).

**Table 5:** Comparison of microaneurysm (MA) counts before and after treatment.

Parameter	Before treatment	1 Month post-treatment	3 Months post-treatment	6 Months post-treatment
n=46	84.60 ± 20.74	78.70 ± 18.66	72.71 ± 20.53	63.06 ± 17.08
t-value		1.4326	2.7633	5.4360
P-value		0.1554	0.0069	<0.001

**Table 6:** Comparison of the number of patient with reduced hard exudate (HE) area before and after treatment.

HE area category	Before treatment	1 Month post-treatment	χ2	P value	3 months post-treatment	χ2	P value	6 Months post-treatment	χ2	P-value
No HE	1	1	0.7156	0.8695	2	3.5766	0.3110	2	11.0895	0.0113
<0.5mm^2^	2	4			7			9		
0.5mm^2^-2.5mm^2^	18	17			16			24		
>2.5mm^2^	25	24			21			11		

**Table 7:** Comparison of neovascularization (NV) area (mm²) before and after treatment.

Parameter	Before treatment	1 Month post-treatment	3 Months post-treatment	6 Months post-treatment
n=46	0.98 ± 0.17	0.17 ± 0.10	0.17 ± 0.09	0.17 ± 0.08
t-value		28.6425	28.9329	29.5133
P-value		<0.001	<0.001	<0.001

### 3.5 Adverse events

No serious systemic or ocular adverse events were reported. Three patients experienced mild subconjunctival hemorrhage following injection, which resolved spontaneously within 1 week without specific intervention.

## 4 Discussion

This study systematically evaluated the efficacy of intravitreal faricimab combined with PRP in PDR patients with DME, with particular focus on changes in retinal venous diameter—a key indicator of microvascular homeostasis. The results indicated that this combination not only significantly improved BCVA and alleviated macular edema, but also for the first time observed a statistically significant reduction in retinal venous diameter at the 6-month follow up, indicating that this combination therapy may reverse DR-associated retinal venous alterations.

The continuous improvements in BCVA and significant reductions in CFT are consistent with prior studies (11). In the phase III YOSEMITE and RHINE trials, faricimab administered every 8 weeks or using a treat-and-extend regimen (up to every 16 weeks) demonstrated non-inferiority to aflibercept 2.0 mg in terms of BCVA change from baseline to year 1 (week 48) (8, 12, 13). Moreover, faricimab was associated with greater reductions in central foveal thickness and retinal fluid volume compared to aflibercept, with sustained visual and anatomical improvements through the second year. Faricimab was well tolerated, with a safety profile comparable to aflibercept at both 1- and 2-years (8, 13). A short-term real-world study in Japan also confirmed that treatment-naïve DME patients showed significant BCVA improvement and marked alleviation of macular morphological abnormalities after 6 months of faricimab therapy (14). Collectively, these findings support dual functional and anatomical benefits of faricimab in PDR patients with concomitant DME.

Notably, significant reduction in retinal venous diameter was observed only at 6 months, with no significant changes at earlier time points, indicating that this effect may rely on sustained dual-pathway inhibition. This observation is consistent with our previous 2-year retrospective study in high-risk PDR patients, in which a comparable reduction in retinal venous diameter was identified 12 months after treatment with aflibercept combined with PRP (15). During the progression of DR, pericyte apoptosis, basement membrane thickening, and endothelial dysfunction collectively contribute to retinal ischemia and hypoxia, subsequently leading to venous beading and compensatory enlargement of the vascular lumen (16). Thus, alterations in retinal venous diameter may reflects mitigation of retinal microvascular ischemia and partial restoration of vascular homeostasis.

The Ang-2/Tie2 axis plays a pivotal role in regulating endothelial-pericyte interactions and vascular stability. Under physiological conditions, pericyte-derived Ang-1 activates endothelial Tie2 receptors, thereby reinforcing endothelial barrier integrity and suppressing inflammation. Within the DR microenvironment, hypoxia and inflammatory stimuli induce substantial endothelial release of Ang−2, antagonizing Ang-1/Tie2 signaling and ultimately resulting in pericyte dropout, aggravated vascular leakage, and neovascularization (17). Faricimab, by simultaneously inhibiting Ang-2 and VEGF-A, both of which promote inflammation and vascular permeability, may synergistically preserve pericytes and restore endothelial integrity, thereby contributing to retinal venous structural remodeling.

The delayed improvement in retinal venous diameter observed in this study may be attributed to the slower process of Ang-2-mediated vascular remodeling compared with VEGF-driven leakage inhibition. Furthermore, impaired autoregulation of retinal vessels is associated with reduced vascular tone and structural alterations in the vessel wall. Additionally, vascular endothelial dysfunction may compromise endothelium-dependent vasodilation (18). Focusing laser irradiation on leaking capillary areas is an effective way to reduce swelling by inducing vascular remodeling and reducing hydrostatic pressure (19).Thus, the improvement in venous diameter observed with faricimab over a relatively shorter duration underscores its distinct mechanistic advantage over conventional anti-VEGF agents. However, further validation through larger sample sizes, longer follow-up, and prospective multicenter studies remains necessary.

MAs are hallmark early microvascular lesions of DR, histologically characterized by pericyte loss, endothelial cell proliferation, and apoptosis, with their quantity closely reflecting disease activity (20). This study demonstrates that MA began to decrease significantly at 3 months, with a 25.5% reduction at 6 months, indicating that faricimab effectively curbs progressive microvascular injury. A 2-year study conducted in Taiwan reported that both aflibercept and ranibizumab significantly reduce MA counts (21). A subgroup analysis of the CLARITY study demonstrated that complete regression of NV was achieved by week 12 in all eyes receiving anti-VEGF combined with PRP therapy within the 52-week observation period (22). Preventing further vitreous hemorrhage and leakage remains a primary therapeutic goal in managing NV, which aligns with the findings of our team’s previous research. Following anti-VEGF therapy, significant regression of NV can be observed (23).

HE primarily consists of lipids and proteins leaked from dilated capillaries and microaneurysms. The ETDRS reported that persistent central macular edema may progress to subretinal fibrosis, resulting in irreversible vision loss (24). Previous studies have demonstrated a significant reduction in HE following anti-VEGF therapy (25), and our previous research yielded similar results (23). In the present study, HE was significantly reduced at 6 months after treatment. We posit that this reduction may be attributed to the marked decrease in MA following anti-VEGF therapy, improvement in vascular permeability and partial restoration of lipid metabolism. The decrease in HE area following anti-VEGF treatment may be associated with the reduction of MA and the recovery of retinal vascular health following ischemia and hypoxia.

This study has several limitations. First, its retrospective design may introduce selection and information biases. Due to the retrospective design and incomplete availability of systemic medical history and medication records, our baseline characterization is limited to the variables presented in **Table 1**. Future prospective studies should include a more detailed assessment of comorbidities and concurrent medications.Second, the sample size is relatively small. The lack of a monotherapy or untreated control group limits our ability to definitively attribute the observed outcomes specifically to the combined regimen or to quantify its incremental advantage over individual components. Thus, while our pre- and post-treatment comparisons provide supportive evidence, the results should be interpreted as preliminary. Future randomized controlled trials with appropriate control arms (e.g., monotherapy alone or placebo) are necessary to establish the specific contribution and superiority of the combined treatment.Third, although retinal venous diameter was measured using standardized formula, the measurement is constrained by FFA image quality and the inherent subjectivity of manual interpretation. Future prospective, multicenter, large-sample randomized controlled trials incorporating artificial intelligence-assisted image analysis are warranted to enhance measurement precision and the robustness of the findings.

In conclusion, intravitreal faricimab combined with PRP demonstrates favorable efficacy and safety in PDR patients with DME, including improvements in BCVA, reductions in CFT, decreases in MA counts and NV area, resolution of HE deposition, and promotion of structural improvement in retinal venous diameter. Although its long-term clinical benefits require further validation through extended follow-up, the current evidence provides strong support for this combination regimen as an optimized therapeutic strategy for PDR with DME.

## Data Availability

The original contributions presented in the study are included in the article/supplementary material. Further inquiries can be directed to the corresponding authors.
